# Correlation Coefficient Based Optimal Vibration Sensor Placement and Number

**DOI:** 10.3390/s22031207

**Published:** 2022-02-05

**Authors:** Geon-Ho Shin, Jang-Wook Hur

**Affiliations:** Mechanical Engineering (Department of Aeronautics, Mechanical and Electronic Convergence Engineering), Kumoh National Institute of Technology, Gumi 39177, Korea; 20216052@kumoh.ac.kr

**Keywords:** automatic storage, finite element analysis, fisher information matrix, modal mass, mode shape

## Abstract

Vibration sensors are mostly used for fault diagnoses of machines or structures. If more sensors are applied, more accurate fault diagnosis is possible. However, it will obviously cost more. There are many approaches to optimize the number and installation location/point of vibration sensors for more efficient fault diagnosis. Existing methods require a great deal of computational throughput for optimization when considering many mode frequencies with points where vibration sensors are likely to be installed. This paper proposes a practical way of optimizing the sensor installation point considering many mode frequencies with lots of places for sensor installation. FEA was conducted to identify displacement values of each frequency in the candidate points. Then, correlation coefficients were applied to the FEA result to optimize the installation location and number of vibration sensors. Taking into account cases where the number of sensors has been set by users, FIM was applied. The correlation coefficient optimized the candidate points where 24,252 vibration sensors were to be installed and reduced this to 10 points. FIM, which was not suitable for directly optimizing sensor locations because it required a lot of computational throughput and was usually applied to evaluate other methods, is now applicable to candidate points that have been reduced by the correlation coefficient. This paper does not draw the best optimal sensor location but presents a way to apply to large-scale or complicated forms with a little computational throughput.

## 1. Introduction

In order to diagnose a failure of machines or structures, a method of collecting and diagnosing data by attaching vibration sensors is widely used [1,2,3]. However, it is expensive to attach the sensors to all necessary portions of the target and it requires a lot of computational throughput for data collection and fault diagnoses with the data collected from the sensors. Thus, it is necessary to install the proper number of vibration sensors that are needed at proper points. The most commonly used method is to extract the mode shape of the target with FEA (finite element analysis); then, an algorithm for optimizing the sensor location is applied to the analysis result drawn from each node. Here, the node positions become the candidate points where sensors will be installed. The best sensor locations are those with the largest FIM (Fisher information matrix) determinant values; FIM determinant values are used to evaluate optimized sensor locations with an algorithm [4,5,6]. FIM determinant values may be directly used to optimize sensor location, but there are too many determinant values to calculate with computers. Therefore, there have been numerous studies on effective optimal sensor placement algorithms and even comparative studies on which algorithm is the most suitable one for optimization. Z. N. Li, J. Tang and Q. S. Li [7] applied four different methods for optimized vibration sensor installation location, such as GA (Genetic algorithm), MSF (modal scale factor), MAC (modal assurance criterion), and similarity of two vibration modes to cantilevers and compared the results. G. Martin, E. Balmes, and T. Chancellier [8] applied four methods for optimizing vibration sensor installation location to drum brake parts, including MAC, CoMAC (coordinate MAC), eCoMAC (enhanced CoMAC), and MACCo (MAC coordinate) and compared the results. C. Yang, R. Ma and R. Ma [4] applied six different methods for optimized vibration sensor installation location, including Efi (effective independence), DPR (driving point residue), ADPR (average DPR), Efi-DPR, EVP (eigenvalue vector product), and MSSP (modal shape summation plot) to satellites and compared the optimization results. D. C. Kammer and M. L. Tinker [5] applied two different methods for optimized vibration sensor installation location in the order of MKE (modal kinetic energy) and Efi3 to a reusable launch vehicle and optimized the sensor location. M. Meo and G. Zumpano [9] applied Efi, Efi-DPR, MKE, EVP (Eigenvalue vector product), NODP (non-optimal drive point) and VM (variance method) to optimize the vibration sensor installation location on bridge structures and compared the results. C. Yang [6] applied a synthetic method by giving a weight on several optimization methods.

Unless actual data are collected from sensors attached to machines or structures with faults, vibration characteristics in the fault state are unknown. Thus, it is necessary to optimize sensor location for fault diagnosis in consideration of the proper amount of mode shapes. Moreover, the proper number of nodes should be arranged to gain reliable FEA results [10,11]. Most studies on algorithms to optimize vibration sensor position are conducted to identify proper sensor location reducing computational throughput; however, they are still insufficient to optimize the position of vibration sensors considering many mode shapes and nodes. In fact, most studies optimize the vibration sensor location with algorithms that are applied to the FEA results drawn considering only a few mode shapes or with candidate points less than 10,000 [4,5,6,7,8,9,12,13,14].

**Figure 1:**
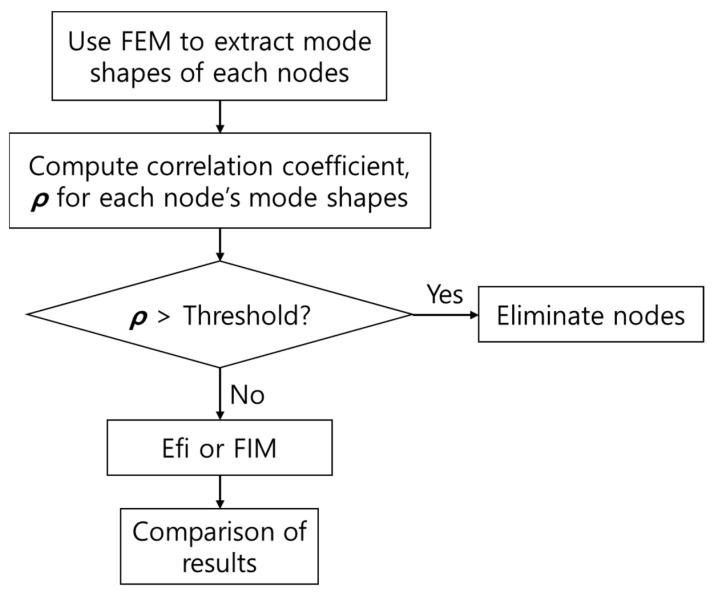
Flowchart.

This paper proposes an algorithm for practically optimizing vibration sensor installation location that is applicable even when there are lots of mode shapes and nodes to consider with less computational throughput when compared to other conventional ways. First, a correlation coefficient was applied to mode shapes of each node drawn from the FEA result. In general, the correlation coefficient is used to build a deep learning-based fault diagnosis model with collected data from vibration sensors [15,16,17]. However, it is used to optimize both the number and location of vibration sensors in the paper, which allows one to produce valid results with less computational throughput. When the number of vibration sensors optimized with the correlation coefficient is larger than the number of sensors retained, the existing algorithms, such as Efi or FIM, can be applied to mode shapes of the optimized sensor points to acquire final sensor points. FIM had not been applicable, as it required a lot of computational throughput, but was applicable here, as many nodes are already eliminated from the candidate group by the correlation coefficient. The FIM results were compared to those of Efi, which has been regarded as the most effective algorithm in several studies [4,9]. The overall procedure adopted for the study is shown in Figure 1 and was applied to an AS/RS (automatic storage and retrieval system), SAR-400, to show the result.

## 2. Theoretical Background

According to the study herein, first, the correlation coefficient is applied to mode shapes of the AS/RS unit acquired from FEA. Then, other existing methods are applied in order to optimize the location and number of vibration sensors.

After generating $S_{0}$ nodes in FEA, a modal analysis to calculate N mode frequencies was conducted and mode shapes at each mode frequency were extracted. The mode shape consists of displacement values of each node calculated at relevant mode frequency and the displacement value is expressed by column vector $d^{i}$ of $S_{0}\times 1$ dimension.

(1) $$d^{i}={\left\{d_{1}^{i},\;d_{2}^{i},\;\ldots ,\;d_{S_{0}}^{i}\right\}}^{T},\;i=1,\;2,\;\ldots ,\;N$$

here, $d_{j}^{i}$ is the modal analysis result, the displacement at jth node from ith mode frequency. Assuming that sensors are all attached to each node, the displacement of all mode frequencies may be expressed as the following vector.

### 2.1. Correlation Coefficient

In general, when developing a deep learning-based fault diagnosis model with data collected from vibration sensors, a correlation coefficient is applied to features extracted from the original data to reduce computational throughput; only one feature among those with high correlation remains, while the rest are eliminated [15,16,17]. Applying correlation coefficients for optimizing the number and location of sensors started from this idea. When the correlation coefficient is applied to mode shapes of each node drawn from FEA results, only one node among nodes with correlated mode shapes remains; the rest of the nodes are eliminated from the installation point candidates, which optimizes the location and number of vibration sensors. When sensors are attached to the positions of nodes with correlated mode shapes, the data collected by each sensor can be determined to be similar. It gets more effective when collecting data with a fast sample rate or a long sample collection period. This is because the mode shape values drawn from FEA results are far smaller than the actual data variables.

The correlation coefficient represents the dependence of two consecutive variables as a value between +1 and −1. Some representative correlation coefficients include Pearson’s, and Spearman and Kendall’s tau correlations, which are shown in (2–4), respectively [18,19].

(2) $$\rho_{P}=\frac{\sum_{i=1}^{N}\left\{\left(d_{j_{1}}^{i}-mean\left(d_{j_{1}}\right)\right)\left(d_{j_{2}}^{i}-mean\left(d_{j_{2}}\right)\right)\right\}}{\sqrt{\sum_{i=1}^{N}{\left(d_{j_{1}}^{i}-mean\left(d_{j_{1}}\right)\right)}^{2}\sum_{i=1}^{N}{\left(d_{j_{2}}^{i}-mean\left(d_{j_{2}}\right)\right)}^{2}}}$$

(3) $$\rho_{S}=\frac{\sum_{i=1}^{N}\left[\left\{rank\left(d_{j_{1}}^{i}\right)-mean\left(rank\left(d_{j_{1}}\right)\right)\right\}\left\{rank\left(d_{j_{2}}^{i}\right)-mean\left(rank\left(d_{j_{2}}\right)\right)\right\}\right]}{\sqrt{\sum_{i=1}^{N}{\left\{rank\left(d_{j_{1}}^{i}\right)-mean\left(rank\left(d_{j_{1}}\right)\right)\right\}}^{2}\sum_{i=1}^{N}{\left\{rank\left(d_{j_{2}}^{i}\right)-mean\left(rank\left(d_{j_{2}}\right)\right)\right\}}^{2}}}$$

(4) $$\rho_{K}=\frac{\sum_{i=1}^{N}\sum_{k=1}^{N}sgn\left(d_{j_{1}}^{i}-d_{j_{1}}^{k}\right)sgn\left(d_{j_{2}}^{i}-d_{j_{2}}^{k}\right)}{N\left(N-1\right)}$$

where: $sgn\left(x\right)=\{\begin{array}{ll} 1 & if\;x>0\\ 0 & if\;x=0\\ -1 & if\;x<0 \end{array}$.

While existing methods to be described later simultaneously consider the mode shapes of all nodes, the correlation coefficient has much less computational throughput, since the mode shapes of two points are considered in a single repeated calculation. In addition, the number of iterations itself is much lower than other methods, because the nodes with correlation coefficient values above the threshold are excluded at once. If the number of nodes excluded by the correlation coefficient is lower than intended, it can be re-determined by changing only the threshold, so it requires less computational throughput than the existing method.

### 2.2. Fisher Information Matrix

Since many nodes are excluded from the candidate points where sensors are likely to be installed with the correlation coefficient, FIM, which was not used as it required a great deal of computational throughput, is applied.

(5) $$y=\left[d^{1}\;d^{2}\;\ldots \;d^{N}\right]q+w=Dq+w$$

here, $q={\left\{q_{1},\;q_{2},\;\ldots ,\;q_{N}\right\}}^{T}$ that consists of $q_{j}\left(j=1,\;2,\;\ldots ,\;N\right)$ refers to the contribution on y. w is the noise related to the sensor measurement and D is the matrix comprised of displacements estimated with the modal analysis. Vector q indicates the contribution estimated from each mode frequency and the estimate error covariance matrix is expressed as below.

(6) $$X=E\left[\left(q-\hat{q}\right){\left(q-\hat{q}\right)}^{T}\right]={\left[D^{T}R^{-1}D\right]}^{-1}$$

here, $\hat{q}$ is the estimate of q, E is the expectation operator and R is the covariance matrix of noise. The result of (6) is the inverse matrix of FIM Q, which can be defined as below.

(7) $$Q=D^{T}R^{-1}D$$

Maximizing Q results in minimizing X and, in turn, the nearest q’s estimate, $\hat{q}$, can be calculated. Therefore, vibration sensors should be installed at points where Q is maximized. FIM can be replaced by the following equation.

(8) $$Q=D^{T}R^{-1}D=\left(D^{T}R^{-1/2}\right)\left(R^{-1/2}D\right)={\bar{D}}^{T}\bar{D}$$

here, $\bar{D}=R^{-1/2}D$ is the displacement matrix with noise taken into consideration. If noise w shows no correlation with sensors and sensors possess the same noise characteristics, noise does not influence the determining of the sensor location. Thus, the Fisher information matrix may be simplified as follows [12].

(9) $$Q=D^{T}D$$

Parameters to maximize Q include trace, condition number and determinant values. In particular, the determinant |Q| holds the largest value when the estimate is the best. Therefore, it was applied for this study [13].

### 2.3. Effective Independence

**Figure 2:**
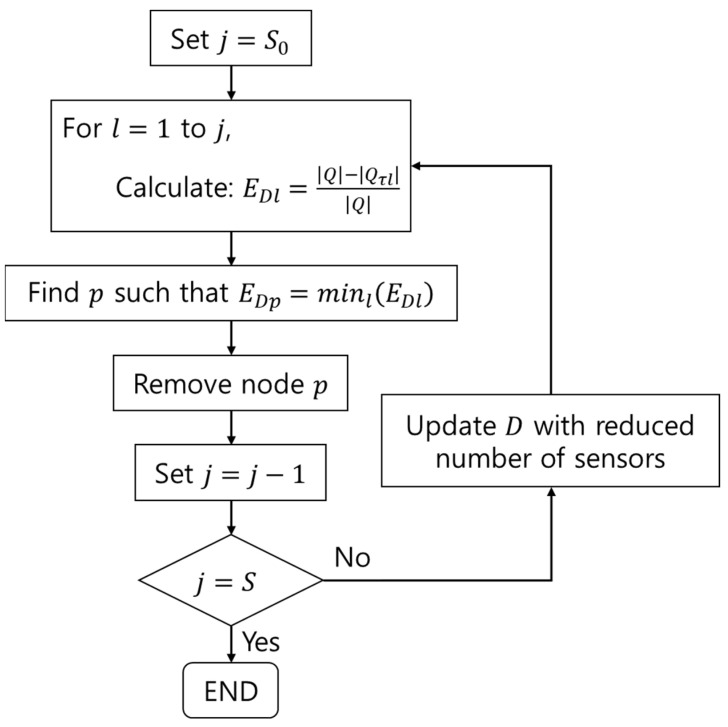
Process of Efi.

Efi for lth node, $E_{Dl}$, can be expressed in FIM as shown in (10).

(10) $$E_{Dl}=\frac{\left|Q\right|-\left|Q_{\tau l}\right|}{\left|Q\right|}$$

here, $Q_{\tau l}$ expresses the FIM with lth node deleted from the candidate point. In other words, $E_{Dl}$ shows how the determinant of FIM changes when the lth node is removed from the candidate point. Hence, the most efficient sensor installation point can be identified when the algorithm repeatedly removes the node at which $E_{Dl}$ represents the minimum value from the candidate points until the number of nodes is equivalent to the number of vibration sensors that users want to remain. Here, |Q| is recalculated with the displacement of nodes that have been decreased in the previous step. The overall calculation steps are shown in Figure 2; S indicates the number of vibration sensors that users want [13,14].

## 3. Numerical Example

### 3.1. Target Shape and Mechanical Properties

**Figure 3:**
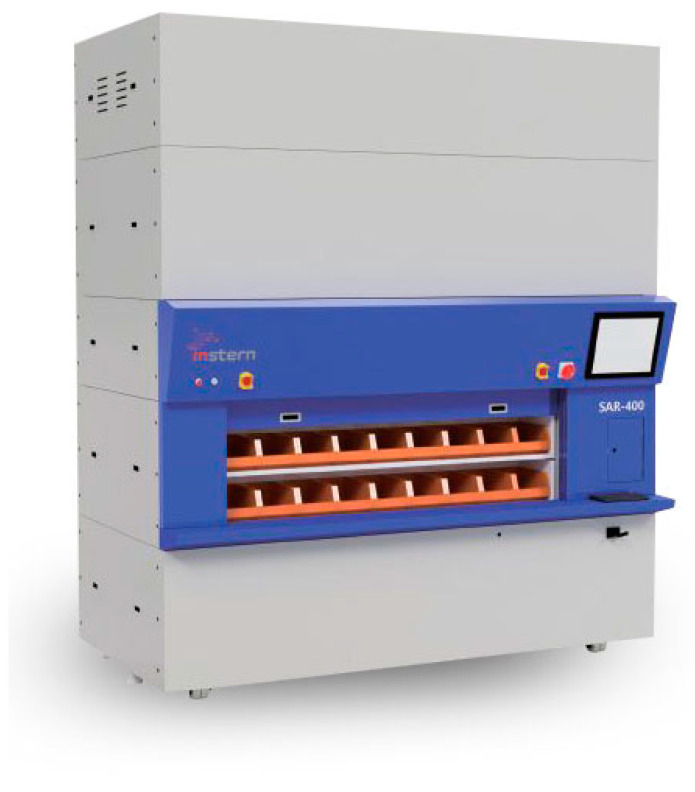
AS/RS configuration.

In this section, we will examine the location and number of vibration sensors optimized by the correlation coefficient, FIM and Efi, stated in Section 2 for the AS/RS unit fault diagnosis. An AS/RS unit is a device designed to bring out items that users are searching for in a list of loaded items without having to move around the actual warehouse to take out articles. The shape of the AS/RS unit SAR-400 that the study adopted is shown in Figure 3 and is a rotary type of device in which the pallets containing articles rotates up and down. In the SAR-400, parts made of pre-coated steel sheets and stainless steel are connected to the aluminum profiles and brass hexagonal posts. The mechanical properties of materials used for analysis are described in Table 1 [20].

### 3.2. Boundary Conditions and Meshing

**Figure 4:**
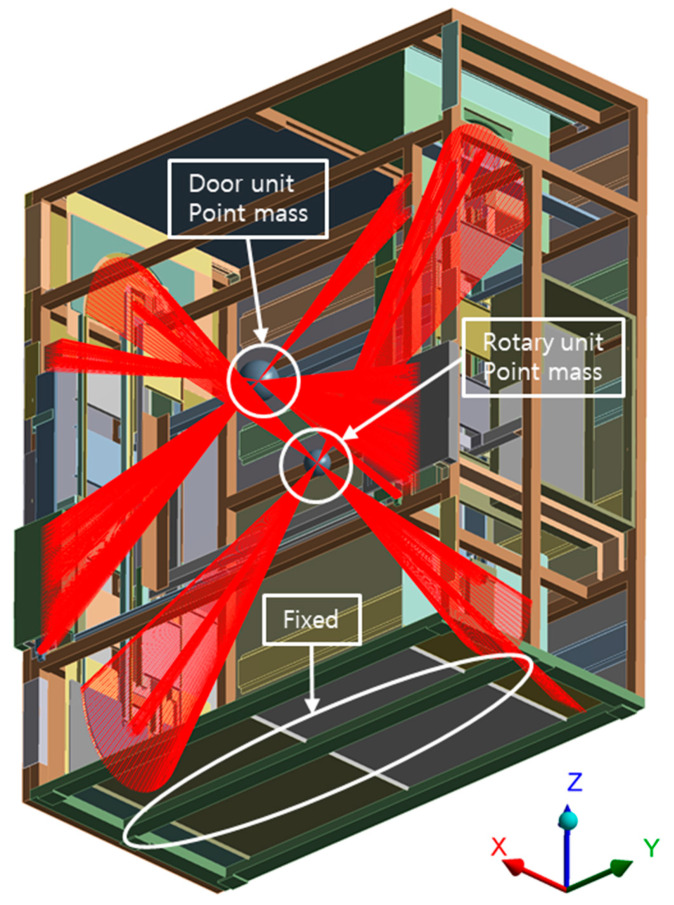
Boundary Conditions.

Nodes generated at the meshing step become the candidate points where vibration sensors may be installed. Thus, the study conducted an analysis with point mass replacing moving parts because it is meaningless to calculate the displacements for moving parts in which sensors cannot be attached and it requires a great deal of computational throughput. The moving parts are mainly grouped into door parts and rotating parts; the mass points that replace those parts are shown in Table 2. In addition to this, the boundary condition of FEA is illustrated in Figure 4.

Moving parts had all been replaced with point mass; there were no dynamic components inside the SAR-400 modeling. Thus, bonded contact conditions were added to all contact surfaces so that the cut surfaces or contact surfaces of the components did not separate or rub against each other. In addition, augmented Lagrange was adopted as the formulation of the contact conditions. Augmented Lagrange is a contact algorithm based on the penalty method and features an increased probability of convergence by adding a Lagrange multiplier to pure penalty. It can also calculate the analysis results of all surfaces with contact conditions. Gauss point detection was used to detect contact points, which increases the compatibility of contact surfaces with the same curvature [21,22].

**Figure 5:**
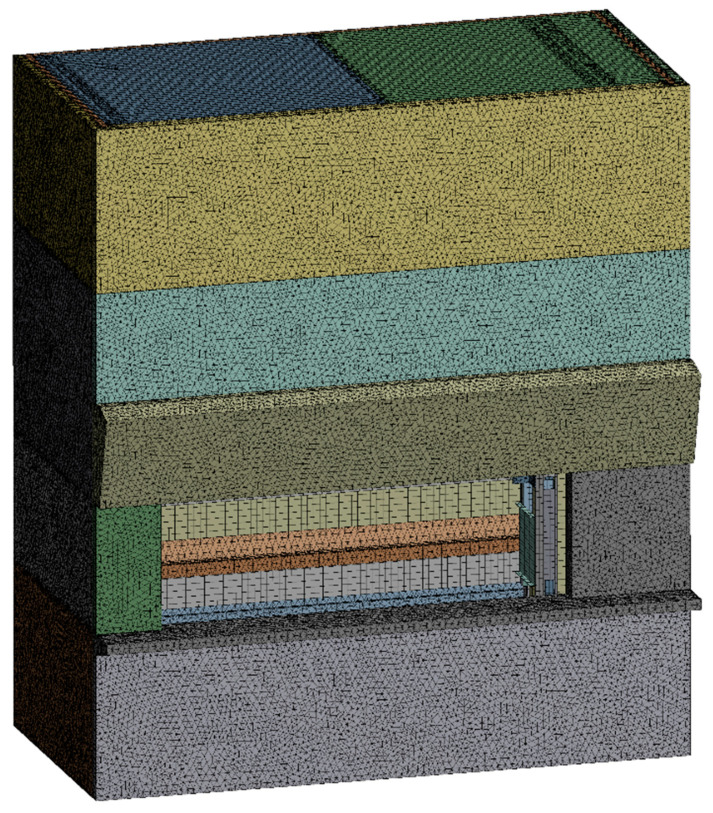
Mesh Division.

Since the node point is the point where a vibration sensor will be installed, the element size is the interval of sensors. Thus, the study assumed that the sensor size was 25 mm and set the average element size as 25 mm to prevent sensor overlaps. The divided element shape is illustrated in Figure 5; they are divided into 1,564,172 nodes and 647,369 elements with the application of tetrahedral and hexahedral elements.

### 3.3. Modal Analysis Results

**Figure 6:**
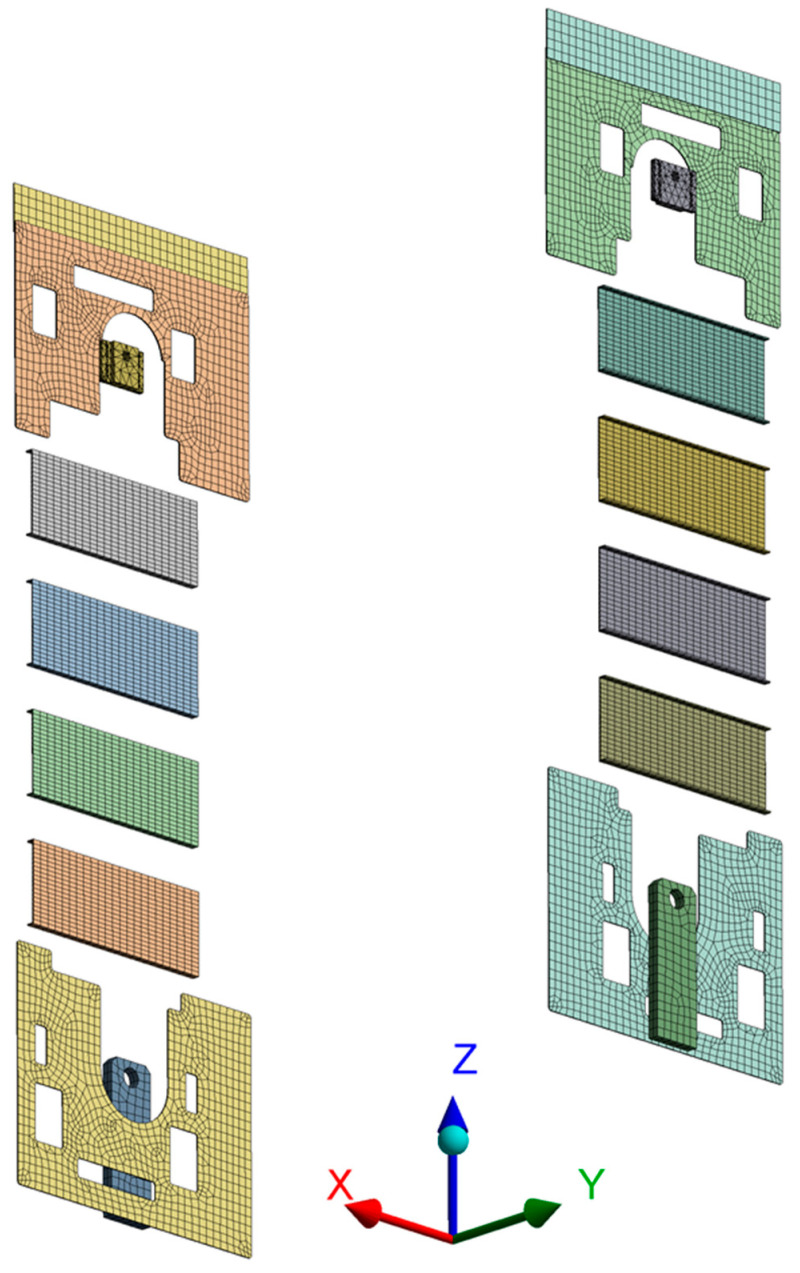
Vibration Sensor Location Candidate.

As shown in Figure 6, the analysis result was drawn from points where there was enough space to install sensors and was connected to the moving parts. In the process, the candidate points where sensors could be installed were reduced to 24,252.

**Figure 7:**
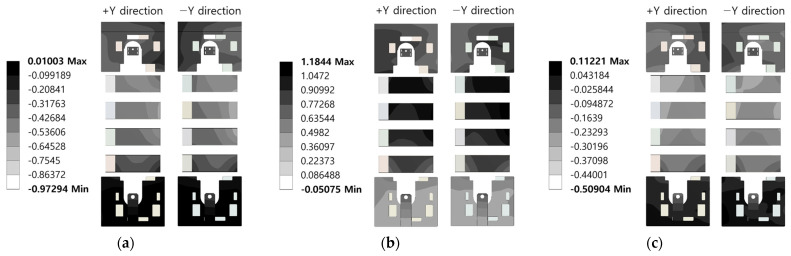
Mode Shapes: (**a**) 25th; (**b**) 26th; (**c**) 35th.

Modal analysis was adopted to estimate the mode frequency and shape of the AS/RS unit. However, it may require a lot of computational throughput when too many modes are extracted. To prevent this phenomenon, M. E. DEMİR [23] and E. TASDELEN [24] extracted mode frequencies until the effective modal mass was more than 90% and 80%, respectively. Here, the effective modal mass indicates the mass of each mode affected in a specific direction [25]. Considering the shape of the components with vibration sensor attachment position candidates and the direction in which they are fastened, the vibration in the *Y*-axis direction possesses the greatest influence on them. Therefore, this study extracted mode frequencies until the effective modal mass in the *Y*-axis direction was more than 80% of the total mass. In total, 302 modes were extracted; they are partially presented in Table 3. In addition, Figure 7 shows the images viewed from the +Y direction of the 25th, 26th, and 35th modes with the highest effective modal mass and from the −Y direction.

### 3.4. Optimal Sensor Placement and Number Results

**Figure 8:**
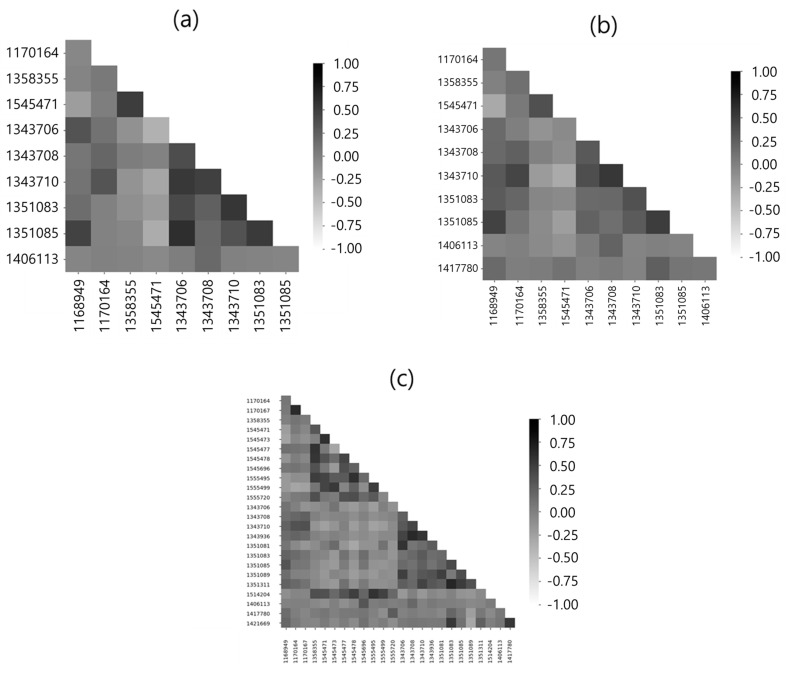
Correlation Coefficient Result: (**a**) Pearson; (**b**) Spearman; (**c**) Kendall tau.

The correlation coefficient compares mode shapes of each node extracted from the FEA result; only one node among the nodes with correlated mode shape remains, while the rest are excluded from the candidate group. Nodes with a correlation coefficient of 0.7 or above were removed from the candidate group, as shown in Table 4; the correlation coefficient estimation of the remaining points is shown in Figure 8. It was found that the proper number of sensors was extracted according to the reference value.

The lowest number of sensors was extracted from optimization with the Pearson correlation coefficient. Thus, this study applies FIM and Efi to the Pearson correlation coefficient optimization result and compares the results.

**Figure 9:**
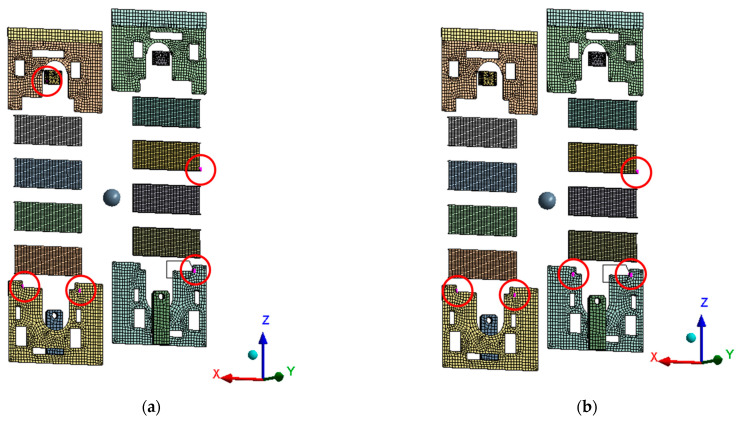
OSP Configuration: (**a**) Pearson-FIM; (**b**) Pearson-Efi.

FIM and Efi were applied to 10 candidate points for sensor installation that had been optimized with the Pearson correlation coefficient, which reduced the number of sensors to 5, as shown in Table 5 and Figure 9. Efi is a method of eliminating sensors one by one and FIM is a method of considering every number of cases until the highest determinant value is calculated when the number of sensors is fixed. Efi is a method that uses FIM for repetitive calculations; however, it was found that the sensor positions applied with Efi were not the same as those applied with FIM. This may be because Efi failed to estimate the global maximum value of |Q| considering all possible cases in the process of eliminating sensor points and, instead, calculated the local maximum.

When Efi is applied directly to 24,252 candidate points, the number of FIM determinant values to be calculated is $\sum_{x=6}^{24,252}x=294,091,863$. In fact, Efi was tried directly, but the computer’s memory could not withstand it due to a lot of computational throughput. When FIM is used directly, the number of FIM determinant values to be calculated is $\left(\begin{array}{c} 24,252\\ 5 \end{array}\right)=6.99\times 10^{19}$, which is more than when Efi is applied directly; thus, it was meaningless to try applying FIM directly because it would have needed to calculate more than when applying Efi directly.

### 3.5. Verification

**Figure 10:**
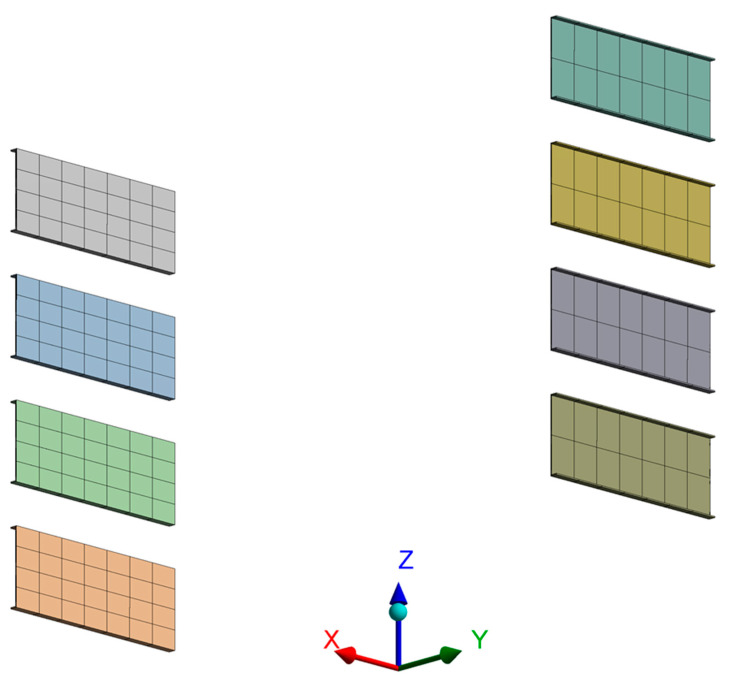
Reduced Vibration Sensor Location Candidate.

To compare and verify the method presented in this paper, as shown in Figure 10, the initial sensor attachment candidate group with reduced nodes was generated to enable direct application of Efi. There were 488 initial sensor attachment candidates in the candidate group; the methods applied here were Efi and Pearson-FIM. FIM was not applicable because it required computational throughput to the extent that they could not be applied even in the reduced candidate group; Pearson-Efi was not applied because it was obvious that it had a lower |Q| than Pearson-FIM. For Efi, we could obtain a local maximum value of |Q| in 488 sensor attachment candidates; since Pearson-FIM derives a global maximum value of the candidate group reduced by the Pearson correlation coefficient, it was meaningful to compare the performance of these two methods.

**Figure 11:**
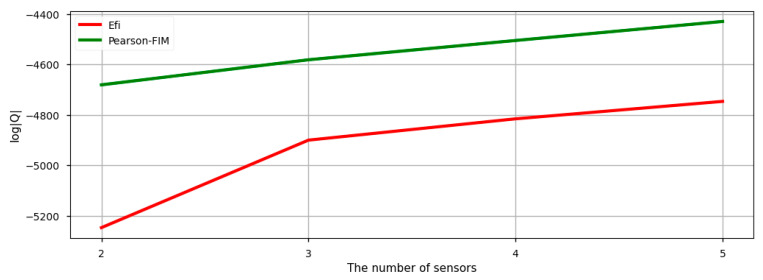
Determinants of Fisher information matrix derived from reduced candidate group.

The application flow presented in this paper was applied to the reduced candidate group as it was; the results are shown in Figure 11. In previous sections, |Q| was not shown because Pearson-FIM obviously displayed higher |Q| than Pearson-Efi; however, this comparative verification indicated which method had higher |Q| and compared its performance. The higher the |Q| value, the higher the mutual independence of the optimized sensor positions, which means that the sensor positions could be confirmed as a combination of sensor positions with higher performance. As a result, it was found that Pearson-FIM possessed higher overall |Q| values than Efi. It has been verified that the method presented in this paper required lower computational throughput and achieved higher performance than Efi.

## 4. Discussion

The correlation coefficient optimizes both the location and the number of sensors. Other algorithms consider mode shapes of all nodes in one repeated calculation, but the correlation coefficient considers only mode shapes of two nodes in one repeated calculation, which results in far less computational throughput. As a result, it was possible to optimize the sensor location with less computational throughput than conventional techniques. The correlation coefficient is disadvantageous in that it cannot optimize after determining the number of sensors; however, if the number of candidates excluded as a result of optimization is less than the intended number, only the threshold can be changed to determine whether to exclude the points. Therefore, even considering recalculating, its computational requirements are small. In addition, even if the number of sensors was accurately determined in advance, other methods can be used in succession; the additional computational throughput that occurs in this case is negligible. For example, the correlation coefficient allows FIM application that was not possible in the past and that reduces computational throughput in Efi. In addition, as a result of comparing the sequential application of correlation coefficients and FIM with Efi, which was judged to have the best performance in several papers [4,9], the method in this paper was found to have lower computational throughput and better performance. However, since this comparative verification was performed in a limited environment, there may be situations in which |Q| of Efi is higher than Pearson-FIM, as, for example, when Efi finds the global maximum value of |Q|. However, even considering this uncertainty, the method in this paper is meaningful in that it is a practical method with much lower computational throughput than other methods. In addition to FIM, these characteristics could serve as a steppingstone for the application of other methods requiring high computational throughput.

Afterwards, comparing data collected in diverse vibration settings after sensors are installed at optimized points is recommended. In addition, if the environment supports it, it would be good to apply FIM directly and to compare it with the method in this paper.

## 5. Conclusions

In this paper, it was confirmed that the location and number of vibration sensors to be attached to the target can be effectively optimized by applying a correlation coefficient to the modal analysis result. The initial candidate points for sensor installation are nodes created at the FEA step; points where sensors cannot be physically installed were primarily excluded. At this step, 24,252 nodes were selected as candidates for sensor attachment. Then, the applied correlation coefficient eliminated nodes, except for one node among nodes with similar vibration characteristics at several mode frequencies from the candidate group. As a result, not only the sensor locations but also the number of sensors were optimized; positions of the sensors decreased from 24,252 to 10. At last, two different methods, Efi and FIM, were applied to the remaining candidate points for sensor installation; the results were compared. FIM is capable of identifying the best installation location, but it requires a lot of computational throughput. Efi may not identify the best sensor installation location, but optimizes the sensor location with reasonable computational throughput. Thus, it is best for users to determine which method to apply after adopting the correlation coefficient. Subsequently, the difference of |Q| between Pearson-FIM and Efi confirmed that |Q| of Pearson-FIM was higher. This may be a coincidence, but Pearson-FIM showed that, unlike Efi, there is no risk of calculating the local maximum when the computational requirements are low.

## Figures and Tables

**Table 1:** Material Properties.

Material	Density (kg/m^3^)	Young’s Modulus (GPa)	Poisson’s Ratio
Aluminum	2770	71	0.33
Brass	8460	103	0.35
Pre-coated steel sheet	7800	204	0.29
SS41	7850	206	0.29
SUS304	8000	197	0.3

**Table 2:** Point Mass.

Definition	Door Unit	Rotary Unit
Mass (kg)	6.927	491.32
Mass moment of inertia X (kg·m^2^)	6.978	496.71
Mass moment of inertia Y (kg·m^2^)	1.341	356.45
Mass moment of inertia Z (kg·m^2^)	5.647	244.15

**Table 3:** Mode Extraction.

Mode	Frequency (Hz)	Modal Mass (%)
1	7.024	0.000
2	8.880	0.000
3	9.010	0.001
⋮		
25	24.621	19.475
26	24.986	31.647
⋮		
33	27.081	2.207
⋮		
35	27.551	4.849
⋮		
82	47.551	4.353
⋮		
122	61.446	1.359
⋮		
262	109.81	1.028
⋮		
300	123.14	0.017
301	123.30	0.291
302	123.39	0.057
Total		80.051

**Table 4:** Correlation Coefficient Results.

Correlation Coefficient	Threshold	The Number of Sensors
Pearson	0.7	10
Spearman		11
Kendall tau		25

**Table 5:** OSP Results.

The Number of Sensors	Node Numbers		Same Node Numbers
	Pearson-FIM	Pearson-Efi	
2	1,358,355, 1,406,113	1,351,085, 1,406,113	1
3	1,343,710, 1,358,355, 1,406,113	1,351,083, 1,351,085, 1,406,113	1
4	1,343,710, 1,351,085, 1,358,355, 1,406,113	1,343,710, 1,351,083, 1,351,085, 1,406,113	3
5	1,343,708, 1,351,083, 1,351,085, 1,358,355, 1,406,113	1,343,708, 1,343,710, 1,351,083, 1,351,085, 1,406,113	4

## Data Availability

Not applicable.
